# Public health professionals' perceptions toward provision of health protection in England: a survey of expectations of Primary Care Trusts and Health Protection Units in the delivery of health protection

**DOI:** 10.1186/1471-2458-6-297

**Published:** 2006-12-07

**Authors:** Paul A Cosford, Mary O'Mahony, Emma Angell, Graham Bickler, Shirley Crawshaw, Janet Glencross, Stephen S Horsley, Brian McCloskey, Richard Puleston, Nichola Seare, Martin D Tobin

**Affiliations:** 1 Directorate of Public Health, East of England Strategic Health Authority, Cambridge, UK; 2 Local and Regional Services, Health Protection Agency, London, UK; 3 Department of Health Sciences and Genetics, University of Leicester, Leicester, UK; 4 Directorate of Public Health, East Midlands Strategic Health Authority, Nottingham, UK; 5 Local and Regional Services, Health Protection Agency, Leicester, UK; 6 Directorate of Public Health, Northamptonshire Primary Care Trust, Kettering, UK; 7 Healthcare Workforce Deanery, East Midlands Strategic Health Authority, Nottingham, UK

## Abstract

**Background:**

Effective health protection requires systematised responses with clear accountabilities. In England, Primary Care Trusts and the Health Protection Agency both have statutory responsibilities for health protection. A Memorandum of Understanding identifies responsibilities of both parties, but there is a potential lack of clarity about responsibility for specific health protection functions. We aimed to investigate professionals' perceptions of responsibility for different health protection functions, to inform future guidance for, and organisation of, health protection in England.

**Methods:**

We sent a postal questionnaire to all health protection professionals in England from the following groups: (a) Directors of Public Health in Primary Care Trusts; (b) Directors of Health Protection Units within the Health Protection Agency; (c) Directors of Public Health in Strategic Health Authorities and; (d) Regional Directors of the Health Protection Agency

**Results:**

The response rate exceeded 70%. Variations in perceptions of who should be, and who is, delivering health protection functions were observed within, and between, the professional groups (a)-(d). Concordance in views of which organisation should, and which does deliver was high (≥90%) for 6 of 18 health protection functions, but much lower (≤80%) for 6 other functions, including managing the implications of a case of meningitis out of hours, of landfill environmental contamination, vaccination in response to mumps outbreaks, nursing home infection control, monitoring sexually transmitted infections and immunisation training for primary care staff. The proportion of respondents reporting that they felt confident most or all of the time in the safe delivery of a health protection function was strongly correlated with the concordance (r = 0.65, P = 0.0038).

**Conclusion:**

Whilst we studied professionals' perceptions, rather than actual responses to incidents, our study suggests that there are important areas of health protection where consistent understanding of responsibility for delivery is lacking. There are opportunities to clarify the responsibility for health protection in England, perhaps learning from the approaches used for those health protection functions where we found consistent perceptions of accountability.

## Background

Are responsibilities for health protection in England clear? One of the consequences of the 2002 changes to NHS structures was a change in the organisations, and in some cases the individuals, responsible for health protection. Primary Care Trusts (PCTs) took on the previous roles of District Health Authorities, which had been defined in the 1977 National Health Service (NHS) Act [1]. These included the duty to promote health, to provide services and to prevent and treat illness. Guidance to the health service identified these as including "arrangements for the control of communicable disease and infections, and for dealing with the health aspects of non-communicable environmental hazards" [2].

**Figure 1:**
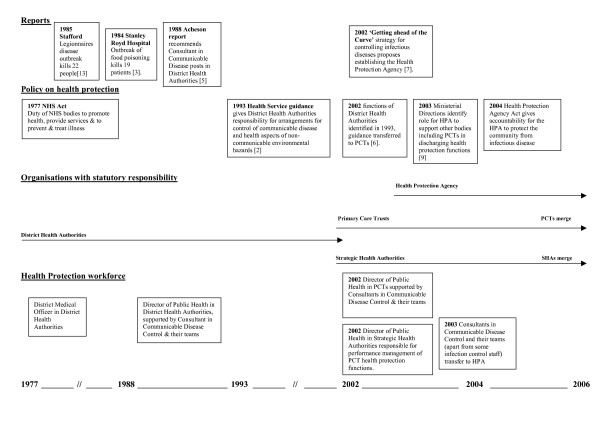
Health Protection Responsibilities in England.

Figure 1 shows how health protection responsibilities have developed in England since 1977. It identifies key reports and consultations, national policy developments, and the organisations and workforce responsible for health protection. In the period up to 2002, responsibility for health protection for any geographical area lay within a single organisation, the District Health Authority. Their functions were clarified and strengthened following two major outbreaks. The first was an outbreak of food poisoning at Stanley Royd Hospital in 1984, which led to the deaths of 19 patients, and the second an outbreak of legionnaires disease in Stafford General Hospital in 1985, which caused the deaths of 22 people. A committee of inquiry into the Stanley Royd outbreak identified a lack of clear accountability for investigation and intervention as a contributory factor [3], and at the time many District Health Authorities did not have adequate capacity to respond to such outbreaks [4]. As a result, a Government review of public health (the Acheson report) led to District Health Authorities investing leadership in the post of the Director of Public Health (DPH) with clear responsibility for public health and health protection [5]. This role was further strengthened by the introduction of specialist Consultants in Communicable Disease Control, who led teams working to the DPH, incorporating surveillance, TB, community infection and outbreak control functions.

Following the 2002 reorganisation of the NHS [6], the health protection responsibilities of District Health Authorities transferred to PCTs, which retained the statutory role of Director of Public Health [1]. The specialist health protection workforce initially transferred to PCTs in support of their Directors of Public Health. It had long been recognised, however, that varying approaches had been taken to health protection between Health Authorities, and further dispersal of responsibility across a much larger number of PCTs would be likely to exacerbate this [7]. The Health Protection Agency (HPA) was established a year later, aiming to create a stronger and more unified health protection system, and intending to bring together the specialists involved in health protection in a single organisation with national specialist functions (such as specialist laboratory and epidemiological services, making these more akin to the Centers for Disease Control in the United States) and local health protection teams [8]. This development aimed to provide a national focus and consistency to the delivery of health protection. The specialist health protection workforce largely moved into the HPA, apart from those carrying out community infection control functions who remained within PCTs.

On its establishment in 2003 the HPA was responsible for supporting other bodies, including PCTs, in carrying out their health protection roles [9]. The HPA Act of 2004 also gave it direct responsibility for protecting the community from infectious and non infectious environmental hazards. However, PCTs were still statutorily responsible for arrangements for the control of communicable disease and non-infectious environmental hazards, as defined in the 1993 guidance to the health service [1].

Hence up to 2002 there was a single organisation responsible at a local level for health protection, but there was significant variation across England in health protection practice. After 2004, there was a much greater critical mass within the national HPA and the potential for more national consistency of practice, but at a local level both PCTs and the HPA had statutory responsibilities for health protection. Unless the roles of each organisation were clearly defined, there was the potential for individuals locally to be unclear about their leadership and support roles in carrying out different health protection functions.

Prior to the establishment of the HPA and PCTs, Health Authorities employed teams to provide their specialist health protection functions. These included: consultants in communicable disease control responsible for the full range of health protection functions; infection control and TB control nurses, responsible for infection control in primary and community care facilities and for TB control; surveillance staff responsible for surveillance of notifiable and other important infectious diseases; and administrative support staff. Despite PCTs retaining statutory responsibilities for health protection from 2003, they largely relied on local Health Protection Agency teams (Health Protection Units – HPUs) to provide this service on their behalf. HPUs mainly consist of the specialist staff previously employed by Health Authorities, aside from community infection control nurses who mostly remained with PCTs.

The local arrangements to hold HPUs to account for providing this service are based on a Memorandum of Understanding. This is based on a national template but has no legal or statutory basis, and it is not expected on its own to achieve uniformity of arrangements across England. It simply reflects agreements between the HPA and the local NHS, for example identifying the role of the HPU in monitoring and surveillance to give early warning of outbreaks of infection, and the responsibility of the PCT in community infection control. In the case of a community outbreak, it usually identifies the Director of Public Health as having overall responsibility for managing the response to the outbreak, and the HPU for carrying out most outbreak control functions on their behalf.

Anecdotal evidence suggests that, whilst arrangements often work well, implementation has varied across England, partly reflecting the inherited NHS variation in service provision. This variation may also reflect variation in geographical distribution of public health specialists responsible for health protection as well as wider public health functions[10]. It might also reflect the dispersal of the public health workforce following the 2002 NHS changes, and the skills gaps identified in the wider public health workforce, particularly in health protection[11]. Alongside the fact that both PCTs and the HPA have statutory health protection roles, these factors may add to a lack of clarity about who is responsible for delivering specific health protection functions locally, and a lack of capacity to deliver those functions. If responsibility for delivery is not clear, significant health protection problems could be unresolved with individuals considering the responsibility to lie with others rather than acting themselves. Inquiries into past health protection incidents in different countries have often identified a lack of clarity as to who is responsible as a major contributing factor [3,12,13]. Equally there is widespread international recognition that systematised responses with clear responsibilities for health protection and other healthcare incidents, are required for responses to be effective. This includes the control of infection [14,15], disaster planning [16,17] and patient safety [18].

The purpose of this survey was to assess the extent of variation in the interpretation of health protection arrangements between PCTs and local health protection teams. In particular it aimed to investigate the perceptions of those who are involved in delivery and oversight of health protection as to whose responsibility it is to deliver specific health protection functions, who delivers them in reality, and to identify issues that need urgent attention. Understanding any differences in perceptions can then inform both the development of the Health Protection Agency and of PCTs, particularly as new arrangements develop in the light of current NHS organisational changes[19]. It is also of relevance to judging the adequacy of health protection arrangements in different countries.

## Methods

We undertook a questionnaire survey of key health professionals responsible for health protection in England during 2005.

### Participants

The participants in this study were: (a) Directors of Public Health in Primary Care Trusts; (b) Directors of Health Protection Units within the Local and Regional Services Division of the Health Protection Agency; (c) Directors of Public Health/Medical Directors in Strategic Health Authorities and; (d) Regional Directors of the Health Protection Agency [see Additional File 1 for further details of sampling frame].

### Questionnaire design, pilot and mailing

A steering group, comprising representatives of each of the four participant categories listed above, designed the study and managed its implementation. The steering group agreed that the study needed to elicit from the four groups of participants ((a) to (d) above): (i) perceptions of the role of the relevant organisations in delivering a range of key health protection functions and; (ii) perceptions of the confidence in safe delivery of these functions. In order that the study could provide timely feedback for policy makers it was agreed that a questionnaire using categorical responses would be used to elicit this information. Five responses were permitted for (i) above: "PCT alone"; "led by PCT with HPA support"; "led by HPA with PCT support"; "HPA alone" or "other", with an option for respondents to clarify their response using free text. A four point scale was used for (ii) above, with responses ranging from "confident all the time" to "not confident". The steering group chose the health protection functions to include in the survey on the basis that they represented a diverse range of activities that, together, represented a substantial proportion of the health protection functions undertaken in England.

The questionnaire was piloted on a convenience sample of 13 individuals that included all four groups of participants listed above. The aim of the pilot was to highlight ambiguous questions and unforeseen problems with the questionnaire. The steering group considered the findings of the pilot, including the feedback of the pilot participants, and extra questions were inserted to reflect the fact that some health protection responses (such as meningitis contact tracing) might be different depending on whether they were required during normal working hours or outside of these hours.

The revised questionnaire was then sent by post to all participants in the study. A second questionnaire was sent to non-responders four weeks after the initial mailing.

### Analysis

Questionnaire data were analysed using with STATA 9.0 [20], using the individual responder (n = 264) as the unit of analysis. We defined a response as 'concordant' if a respondent's views of who should and who does deliver a particular health protection function described the same lead organisation. Subgroup analyses were undertaken within participant groups (a) to (d) in the responders who provided their job title (n = 260).

See Additional File 1 for further details of the pilot data and approaches to data coding.

## Results

**Figure 2:**
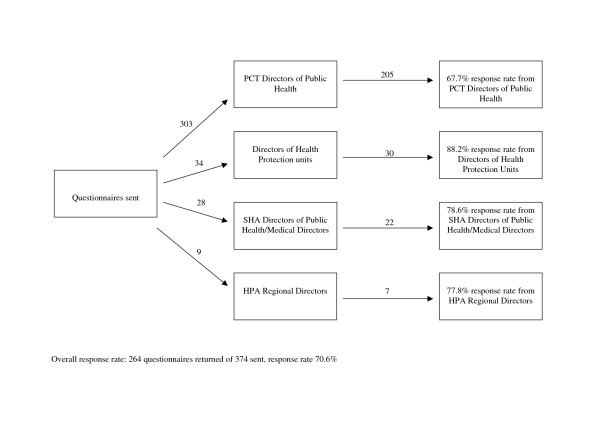
Numbers and Responses by Participant Group.

Questionnaires were sent to 374 individuals in the four participant groups. 264 were returned, an overall response rate of 70.6% (Figure 2).

### Who should be delivering particular health protection functions (Figure 3)?

**Figure 3:**
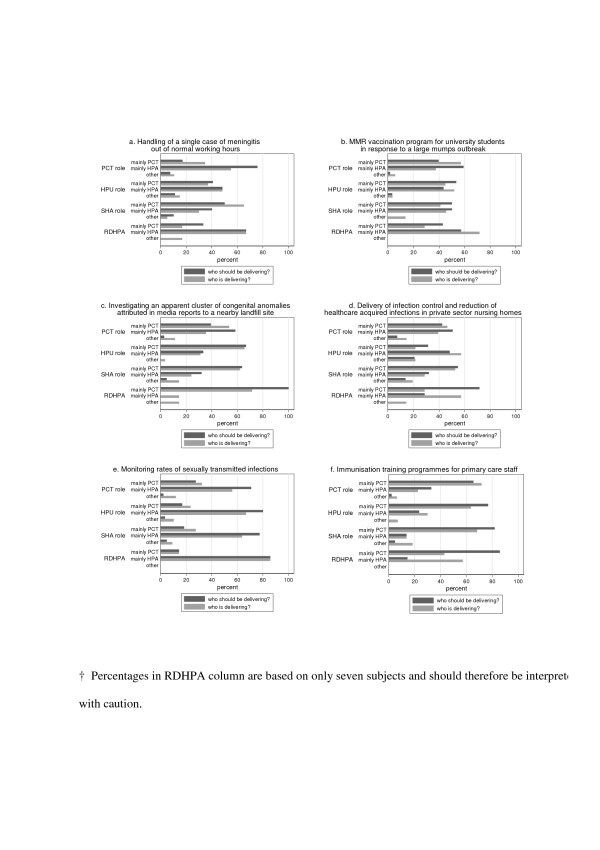
Perceptions of who should be, and who is, delivering health protection functions amongst respondents with PCT, HPU, SHA and RDHPA roles: low concordance response.

There was variation in the expressed views of who should be delivering a particular health protection function among individuals undertaking similar health protection role. For example, of those individuals with a PCT role, 17% stated that the health protection response to a single case of meningitis should be led by the PCT, whereas 76% stated that it should be led by the HPA (Figure 3a, PCT roles, bars with dark shading). Furthermore, there was also variation in expressed views of who should be delivering a particular health protection function *between *groups of professionals undertaking *different *health protection roles. For example, whilst only 17% of individuals with a PCT role stated that the health protection response to a single case of meningitis should be led by the PCT, 41% of individuals with an HPU role believed that this health protection response should be led by the PCT (Figure 3a, PCT and HPU roles, bars with dark shading). Variation, both within and between different professional roles, in the expressed views of who should be delivering was apparent across a diverse range of health protection functions (Figure 3). Although it is not straightforward to identify a single unifying theme for these health protection functions, many of these health protection duties (e.g. Figure 3a,d,e) form a substantial part of the overall health protection function at a local level.

### Who is delivering particular health protection functions (Figure 3)?

We also identified variation in views of who is delivering a particular health protection function among individuals undertaking a similar health protection role. For example, of those individuals with a PCT role, 35% stated that the health protection response to a single case of meningitis was led by the PCT, whereas 55% stated that it was led by the HPA (Figure 3a, PCT role, bars with light shading). In addition, we found variation in views of who *is *delivering a particular health protection function between groups of professionals undertaking *different *health protection roles. For example, 65% of individuals with an SHA role stated that the health protection response to a single case of meningitis was led by the PCT; in contrast, only 35% of individuals with a PCT role expressed this view (Figure 3a, SHA role and PCT role, bars with light shading).

### Is there concordance between views of who should, and who is, delivering health protection?

The concordance of respondents' views of who should and who is delivering each of the 18 health protection functions included in the survey is summarised in Table 1. Note that a response is defined as 'concordant' if a respondent's views of who should and who does deliver a particular health protection function described the same lead organisation. The six health protection functions with the lowest concordance (range 75 – 80%) are included in Figure 3; those with intermediate concordance (range 83 – 90%) and high concordance (range 90 – 98%) are shown in the additional files [see Additional Files 2 and 3 respectively].

### Confidence in the safe delivery of health protection

The percentage of respondents expressing that they felt confident most or all of the time in the safe delivery of particular health protection functions (Table 2) ranged from 51% (infection control in private sector nursing homes) to 97% (handling of a single meningitis case during normal working hours). Mean confidence levels (across all eighteen health protection functions) were 79% in respondents with a PCT role; 76% in respondents with an HPU role; only 68% in respondents with an SHA role and; 73% in Regional Directors of the Health Protection Agency. There was a strong correlation between the concordance of respondents' views of who should and who is delivering each of the 18 health protection functions and the confidence in the safe delivery of these functions (r = 0.65, P = 0.0038).

### Health protection functions of concern

Whilst there is no obvious threshold at which we should, using our data, identify health protection functions that raise concern about the clarity and safety of their delivery, there are a number of health protection functions with low expressed concordance (≤80%) and low expressed confidence (≤80%) (Tables 1 &2). These were: delivery of an MMR vaccination programme for university students in response to a large mumps outbreak; investigating an apparent cluster of congenital anomalies attributed in media reports to a nearby landfill site; delivery of infection control and reduction of healthcare acquired infection in private sector nursing homes; monitoring rates of sexually transmitted infections; and immunisation training programs for primary care staff. These health protection functions are either proactive, or permit a short period of planning before mounting a response (although a rapid response to public concerns and media enquiries may be needed). We highlight immunisation training programs for primary care staff as an area where suboptimal delivery of the training programs may have little or no measurable short-term impact on the success of immunisation programs; conversely, by the time it noticeably impacts on the success of immunisation programs it may take years to correct.

Conversely, some health protection functions which are infrequently required, but for which a complex multi-agency response is immediately required (such as responding to a factory fire with a potential release of harmful chemicals) had high levels of concordance (91% in normal hours; 86% outside normal hours) and relatively high levels of confidence (82% in normal hours; 72% outside normal hours), especially considering that most of the professionals surveyed would never have been called on to respond to such an incident.

## Discussion

### Main findings of this study

For twelve of the eighteen health protection functions surveyed in this study, there is a high level of concordance (above 80%) in respondent's views as to which organisation should be, and which organisation is, delivering the particular functions. For these functions, the responsibility for health protection appears to be clearly understood. Indeed, the confidence expressed in the safe delivery of these functions also tended to be high.

However, five health protection functions had low levels of expressed concordance (≤80%) and also low expressed confidence (≤80%) (see "*Health protection functions of concern*" above). It is tempting to attempt to find a unifying theme for these functions, but perhaps all one can definitively conclude is that these represent a diverse range of health protection functions. One unifying theme, however, is that these health protection functions do not require an immediate response to an emergency. In contrast, we found relatively high levels of concordance and confidence in other health protection functions, many of which appear to require the specialist expertise of the HPA and some of which require an immediate response to an emergency situation.

This study therefore suggests that there are some areas of health protection where consistent understanding of responsibility for delivery may be lacking. That is, people at the point of delivery may be unclear as to who is in charge, and who is responsible for delivering the function. It is possible that the changes in the organisational structure for health protection may have contributed to the lack of clarity for some health protection functions.

### What is already known on this topic

Reports into previous major health protection incidents have indicated that clarity of responsibility and accountability for delivering health protection functions is of utmost importance in safe and efficient response to health protection incidents [1,3,12,13]. It is also recognised internationally that systematised responses, with clear responsibilities for health protection and other healthcare incidents, are required for responses to be effective. This includes the control of infection [14,15], disaster planning [16,17] and patient safety [18]. The establishment of the post of Consultant in Communicable Disease Control, following the "Acheson report" into the public health function in England [5], aimed to provide clear accountability for this function, with overall responsibility taken by the Health Authority Director of Public Health.

Whilst this system did give clarity of accountability, it also resulted in a dispersed and varied system of health protection expertise across Health Authorities in England, with some isolated practice and the potential for a lack of resilience [1,7]. The Health Protection Agency was established to resolve this by bringing together health protection expertise into a single agency [8]. This aimed to ensure that a well co-ordinated response could be mounted to any health protection incident, with the ability to bring national expertise in any field of health protection to bear quickly as required.

The development of this new system has, however, led to a lack of certainty as to responsibilities for delivery of health protection functions at a local level, in particular between PCTs and Health Protection Units. Although the legislative basis of accountabilities for health protection is based on historical functions of previous organisations and functions of the HPA, there is still lack of certainty about delivery of key activities [1].

### What this study adds

Successful health protection arrangements require absolute clarity as to who is in charge and who delivers which aspects of a health protection response in any particular circumstance. This study shows that, whilst this is the case for many aspects of health protection in England, for many functions there are different perceptions as to the organisation that is responsible. Perceptions differ both within particular organisations (for example, within PCTs) and also between professional groups working in different organisations. Associated with this lack of certainty as to who is responsible, are lower levels of confidence in the safe delivery of these health protection functions. Our findings suggest that action needs to be taken to improve the clarity of delivery of some health protection functions. Perhaps a good starting place for policy makers would be to examine the approaches (such as guidelines) that were previously employed to clarify responsibility for those health protection functions where we found high levels of concordance and confidence. This may provide valuable guidance as to how to improve clarity of responsibility consistently across all health protection functions.

### Limitations of the study

We surveyed the self-reported *perceptions *of the participants, but we did not corroborate these reports with data from actual responses to specific health protection incidents. Clearly, whilst perceptions are important, they do not necessarily reflect whether the actual response to health protection incidents is appropriate and safe. For example, individuals working within PCTs and HPUs could recognise that responsibilities are not always clear, and develop mechanisms to address this locally. The lack of clarity identified in this study does not therefore necessarily relate to lack of safe delivery in practice.

Our survey included eighteen health protection functions, some of which were closely related (for example, in working hours and out of working hours responses to a meningitis case). These eighteen functions do not encompass all health protection functions, nor are they a fully representative sample of all health protection functions or of the workload of any of the four different professional groups surveyed (indeed this would be difficult to assess, since some health protection incidents occur infrequently). Rather, they were chosen by an expert steering committee to illustrate a broad range of scenarios where clarity of responsibility and accountability is important. They reflect mainly reactive rather than proactive functions. Therefore, our report that five of eighteen functions (28%) lacked clarity of responsibility needs to be interpreted with some caution – a higher or lower proportion could have been obtained by a different choice of question. Furthermore, there is no obvious definition of an acceptable level of concordance or confidence in responses to our survey. Whilst we have highlighted health protection functions with levels of expressed concordance and confidence ≤80%), any threshold we could have chosen would have been subjective. We would, therefore, advocate a qualitative rather than a quantitative interpretation of our findings.

Crucially, however, our survey did show important areas where improvements in clarity are required.

Our survey was limited in the depth of information we obtained as it was based on categorical responses. The steering group agreed that this was a necessary design feature for timely completion of the survey given impending organisational change in the National Health Service and Health Protection Agency in England. Therefore, we utilised free text responses only to minimise misclassification of categorical responses. If time had allowed, we might have supplemented our study with an in-depth qualitative study (for example, employing semi-structured interviews) study to develop a thorough understanding of the issues and to explore the reasons for particular expressed views. Indeed, there may be an opportunity to follow up our findings in this way.

It is possible that biases could have been introduced due to non-response or due to coding practices. We did not survey the non-respondents or collect detailed information on their characteristics. However, even in the extreme situation that the characteristics of the responders and non-responders were very different, the bias is very unlikely to have been of sufficient magnitude to materially alter our findings (response rate 70.6% overall; range 67.7% – 88.2% across the organisational groups). Coding practices are also unlikely to have introduced biases of significant magnitude. For example, we did not include "don't know" responses when estimating the proportion of participants who were confident "all or some of the time" in the safe delivery of a health protection function. If such responses actually reflected a lack of confidence then we may have overestimated confidence levels. However, only eight "don't know" responses were encountered over all questions and respondents (maximum of three don't know responses per question), so the extent of any bias introduced is likely to be very small indeed. Again, our findings would not have been materially altered by our coding approach.

Finally, whilst we surveyed professionals involved in health protection in England, we did not extend our study to other countries, where the organisational structure for health protection functions may differ. Our findings are therefore unlikely to be directly generalisable to other countries. However, clarity of accountability of health protection functions is an important issue internationally and the lessons learned from effective implementation of health protection do have an international relevance.

## Conclusion

In the context in which this study was undertaken, that is, related to health protection functions that require timely and complex interventions often involving many agencies and/or components of the NHS, we would argue that clarity of accountability is crucial. However, we found that this clarity was lacking for several important health protection functions. This study indicates that improvements in clarity of responsibility for health protection in England are required. The current re-organisation of PCT and Strategic Health Authority boundaries, alongside changes taking place within the HPA to strengthen their frontline services, provides an opportunity to address these issues. With a move to a smaller number of larger PCTs and possibly Health Protection Units, there is opportunity for greater clarity of arrangements.

## Competing interests

The author(s) declare that they have no competing interests.

## Authors' contributions

PC conceived the study, chaired the steering group, coordinated the study and wrote the manuscript. MDT advised on the study design and piloting, supervised the database design, undertook and supervised data analyses and jointly wrote and revised the manuscript. EA designed the database, administered the questionnaire, undertook data entry and contributed to the manuscript. RP drafted the report of the findings for the National Health Service organisations and contributed to the manuscript. All steering group members assisted in the study design, monitoring and advising on the study's progress, and made contributions to the manuscript. The steering group members were PC, MO'M, EA, GB, SC, JG, SH, BM, RP, NS and MDT. All authors read and approved the final manuscript.

## Pre-publication history

The pre-publication history for this paper can be accessed here:



## Supplementary Material

Additional File 1 Further details of the sampling frame, pilot data and data coding. This file contains further details of the methods of the study, in particular the sampling frame, pilot data and data coding. Click here for file

Additional File 2 Perceptions of who should be, and who is, delivering health protection functions amongst respondents with PCT, HPU, SHA and RDHPA roles: intermediate concordance responses. This figure shows the perceptions of who should be and who is delivering health protection functions for those functions where responses show intermediate levels of concordance between participant groups. Click here for file

Additional File 3 Perceptions of who should be, and who is, delivering health protection functions amongst respondents with PCT, HPU, SHA and RDHPA roles: high concordance responses. This figure shows the perceptions of who should be and who is delivering health protection functions for those functions where responses show high levels of concordance between participant groups. Click here for file

## Figures and Tables

**Table 1:** Percentage of respondents with PCT, HPU, SHA and RDHPA roles expressing concordant* views of who should, and who does, deliver particular health protection functions.

	**Respondent**				
**Health protection function**	**All**	**PCT role**	**HPU role**	**SHA role**	**RDHPA †**
Handling of a single meningitis case during normal working hours	98	98	100	90	100
Handling of a single meningitis case out of normal working hours	78	77	85	80	83
Establish/ensure effective operation of outbreak control team for 3 linked meningococcal cases	94	93	100	91	100
Establish/ensure effective operation of outbreak control team for 4 community legionnaires' cases.	92	89	100	100	100
Improving uptake of MMR vaccine in under 2 year olds.	84	85	83	73	100
Immunisation training programs for primary care staff	80	82	83	68	57
MMR vaccination program for university students in response to a large mumps outbreak	76	75	86	68	86
Infection control & reduction of healthcare acquired infections in primary care premises	86	87	83	86	71
Infection control and reduction of healthcare acquired infections in private sector nursing homes	77	76	82	81	57
Infection control & reduction of healthcare acquired infections in community hospitals	83	82	80	89	83
Development of a local strategy for improving sexual health	93	93	97	91	71
Monitoring rates of sexually transmitted infections	79	77	87	86	100
Organisation of the rota for out of hours health protection arrangements	84	82	93	91	86
Factory fire with release of harmful chemicals during normal working hours	92	93	90	86	86
Factory fire with release of harmful chemicals out of normal working hours.	86	85	96	85	83
Managing an outbreak of tuberculosis in a secondary school.	90	88	90	100	100
Investigating an apparent cluster of congenital anomalies attributed in media reports to a nearby landfill site	75	73	93	70	71
Managing a look-back exercise in response to a surgeon carrying hepatitis C	90	90	83	95	100

* We defined a response as 'concordant' if a respondent's views of who should and who does deliver a particular health protection function described the same lead organisation.

† Percentages in RDHPA column are based on only seven subjects and should therefore be interpreted with caution.

**Table 2:** Percentage of respondents expressing confidence * in the safe delivery of particular health protection functions amongst respondents with PCT, HPU, SHA and RDHPA roles.

	**Respondent**				
**Health protection function**	**All**	**PCT role**	**HPU role**	**SHA role**	**RDHPA †**
Handling of a single meningitis case during normal working hours	97	97	100	91	100
Handling of a single meningitis case out of normal working hours	91	90	96	89	100
Establish/ensure effective operation of outbreak control team for 3 linked meningococcal cases	94	93	97	95	100
Establish/ensure effective operation of outbreak control team for 4 community legionnaires' cases.	92	91	100	90	100
Improving uptake of MMR vaccine in under 2 year olds.	74	77	67	59	71
Immunisation training programs for primary care staff	75	79	70	55	43
MMR vaccination programme for university students in response to a large mumps outbreak	75	77	69	68	57
Infection control & reduction of healthcare acquired infections in primary care premises	67	72	55	41	67
Infection control and reduction of healthcare acquired infections in private sector nursing homes	51	53	57	32	33
Infection control & reduction of healthcare acquired infections in community hospitals	73	78	64	44	71
Development of a local strategy for improving sexual health	69	74	60	50	33
Monitoring rates of sexually transmitted infections	60	61	67	50	43
Organisation of the rota for out of hours health protection arrangements	86	86	87	85	100
Factory fire with release of harmful chemicals during normal working hours	82	83	77	82	86
Factory fire with release of harmful chemicals out of normal working hours.	72	74	69	58	67
Managing an outbreak of tuberculosis in a secondary school.	90	89	93	95	100
Investigating an apparent cluster of congenital anomalies attributed in media reports to a nearby landfill site	60	62	57	52	50
Managing a look-back exercise in response to a surgeon carrying hepatitis C	83	80	92	90	100

* the proportion of respondents stating that they were "confident all of the time" or "confident most of the time".

† Percentages in RDHPA column are based on only seven subjects and should therefore be interpreted with caution.
