# Supplementary material for: Public health professionals' perceptions toward provision of health protection in England: a survey of expectations of Primary Care Trusts and Health Protection Units in the delivery of health protection
Source: BMC Public Health. 2006 Dec 7;6:297. doi: 10.1186/1471-2458-6-297 (PMC1712342; doi:10.1186/1471-2458-6-297)
Supplement: Additional File 1 — Further details of the sampling frame, pilot data and data coding. This file contains further details of the methods of the study, in particular the sampling frame, pilot data and data coding. [file 1471-2458-6-297-S1.doc]

**Additional File 1: Further details of the sampling frame, pilot data and data coding**

**Sampling frame**

We aimed to contact all professionals in England from the following groups: (a) Directors of Public Health in Primary Care Trusts; (b) Directors of Health Protection Units within the Local and Regional Services Division of the Health Protection Agency; (c) Directors of Public Health/Medical Directors in Strategic Health Authorities and; (d) Regional Directors of the Health Protection Agency. The sampling frame was drawn up from the following websites

Group (a): [http://www.nhs.uk/England/AuthoritiesTrusts/Pct/list.aspx](https://securewebmail.le.ac.uk/exchweb/bin/redir.asp?URL=http://www.nhs.uk/England/AuthoritiesTrusts/Pct/list.aspx)

Groups (b) and (d): [http://www.nhs.uk/England/AuthoritiesTrusts/Sha/list.aspx](https://securewebmail.le.ac.uk/exchweb/bin/redir.asp?URL=http://www.nhs.uk/England/AuthoritiesTrusts/Sha/list.aspx)

Group (c): <http://www.hpa.org.uk/lars_homepage.htm>

In each case, the name and address of the relevant professional(s) within each organisation was extracted from the website in March 2005 if the information was available. If this information was incomplete, then we telephoned the organisation to obtain this. Note that these websites currently reflect organisational changes that have taken place since our survey has taken place.

**Pilot study data**

Details of the pilot study are described in the main methods section of the paper. The 13 participants included in the pilot were not re-surveyed. As the majority of questions were unchanged after the pilot study, the pilot data for unaltered questions were incorporated into the dataset for analysis.

**Data coding**

We recoded responses to “mainly PCT”, “mainly HPA” and “other”. We coded a response as “mainly PCT” if the respondent chose the “PCT alone” or the “led by PCT with HPA support” categories or if they responded “other” and their free text response was consistent with the PCT(s) taking the lead role. In this way, we were able to use free text responses to minimise misclassification of the categorical responses. Similarly, we coded a response as “mainly HPA” if the respondent chose the “led by HPA with PCT support” or the “HPA alone” category, or if they responded “other” and their free text response was consistent with the HPA taking the lead role. Where the free text did not clearly identify either the PCT(s) or the HPA as having the lead role for a particular function, the category remained as “other”. Using these definitions, we investigated the concordance of each respondent’s views of who should and who does deliver each health protection function. For questions relating to perceived confidence in the safe delivery of each health protection function, we grouped responses in order to report the proportion of respondents who were confident “most or all of the time”, including only those participants who expressed a view in the denominator.
