# Supplementary material for: Public health professionals' perceptions toward provision of health protection in England: a survey of expectations of Primary Care Trusts and Health Protection Units in the delivery of health protection
Source: BMC Public Health. 2006 Dec 7;6:297. doi: 10.1186/1471-2458-6-297 (PMC1712342; doi:10.1186/1471-2458-6-297)
Supplement: Additional File 3 — Perceptions of who should be, and who is, delivering health protection functions amongst respondents with PCT, HPU, SHA and RDHPA roles: high concordance responses. This figure shows the perceptions of who should be and who is delivering health protection functions for those functions where responses show high levels of concordance between participant groups. [file 1471-2458-6-297-S3.doc]

**Additional file 3:**

**Perceptions of who should be, and who is, delivering health protection functions amongst respondents with PCT, HPU, SHA and RDHPA roles: high concordance responses**

† Percentages in RDHPA column are based on only seven subjects and should therefore be interpreted with caution.
